# Sandwich-structured nanoparticles-grafted functionalized graphene based 3D nanocomposites for high-performance biosensors to detect ascorbic acid biomolecule

**DOI:** 10.1038/s41598-018-37573-9

**Published:** 2019-02-04

**Authors:** Razieh Salahandish, Ali Ghaffarinejad, Seyed Morteza Naghib, Asghar Niyazi, Keivan Majidzadeh-A, Mohsen Janmaleki, Amir Sanati-Nezhad

**Affiliations:** 1 Research Laboratory of Real Samples Analysis, Faculty of Chemistry, Iran University of Science and Technology (IUST), Tehran, 1684613114 Iran; 2 Electroanalytical Chemistry Research Center, Iran University of Science and Technology (IUST), Tehran, 1684613114 Iran; 3 BioMEMS and Bioinspired Microfluidic Laboratory, Department of Mechanical and Manufacturing Engineering, University of Calgary, Calgary, T2N 1N4 Alberta Canada; 4 Center for BioEngineering Research and Education, University of Calgary, Calgary, T2N 1N4 Alberta Canada; 5 Nanothechnology Department, School of New Technologies, Iran University of Science and Technology (IUST), P.O. Box 16846-13114, Tehran, Iran; 6 Biomaterials and Tissue Engineering Department, Breast Cancer Research Center, Motamed Cancer Institute, ACECR, Tehran, Iran

## Abstract

We present a highly sensitive and selective nano-biosensor for rapid, stable and highly reproducible detection of ascorbic acid (AA) in the presence of dopamine, uric acid and other interferences by a three-layer sandwich arrangement of nitrogen-doped functionalized graphene (NFG), silver nanoparticles (AgNPs) and nanostructured polyaniline (PANI) nanocomposite. The enhanced AA electrochemical properties of the NFG/AgNPs/PANI electrode is attributed to the superior conductivity of the NFG-PANI and the excellent catalytic activity of AgNPs. The critical modification of the AgNPs-grafted NFG-PANI coated on very low-cost fluorine doped tin oxide electrode (FTOE) increased the charge transfer conductivity of the electrode (the resistance drops down from 11,000 Ω to 6 Ω). The nano-biosensor was used to accurately detect AA in vitamin C tablets with the recovery of 98%. The sensor demonstrated a low detection limit of 8 µM (S/N = 3) with a very wide linear detection range of 10–11,460 µM, good reproducibility and excellent selectivity performance for AA detection. The results demonstrate that this nanocomposite is a promising candidate for rapid and selective detection of AA in practical clinical samples.

## Introduction

Ascorbic acid (AA) is an effective antioxidant and reducing agent, playing roles in precluding radical-induced disorders like neurodegenerative diseases and cancer^1^. The presence of AA is essential for human metabolic activities particularly for cell differentiation and immune cell function^2^. It is well known that the deficiency of AA may cause scurvy while its excessive intake may lead to stomach convulsion and diarrhea^3^. Moreover, AA is used in biomedical chemistry and diagnosis of food ingredients^4^. Given the health and technological prominence of AA and its low-level concentration in biological and food samples, there is an essential need for the accurate detection of AA for healthcare and food quality and security.

Several techniques such as titrimetric and solid-phase iodine methods^5^, high performance liquid chromatography (HPLC)^6^, colorimetric^7^, and electrophoresis^8^ have been used for AA detection. However, these techniques are complicated, time-consuming and relatively expensive. On the other hand, fluorescence-based nanoclusters^9^, quantum dots^10^, nanoparticles (NPs)^11^, and polymers^12^ have been exploited for AA detection but they have led to false-positive results and restricted selectivity because of the presence of environmental stimulus such as quenchers and cross-contaminations in sandwich assays. Therefore, it is desirable to develop label-free and low-cost AA sensors with high sensitivity and selectivity performance^13^.

Electrochemical techniques have demonstrated label-free response, rapid and low-cost performance, with high sensitivity and selectivity in determination of several different biomolecules^14,15^. However, because of the interference of coexisting electroactive species of AA such as glucose (Glu), dopamine (DA), uric acid (UA), and other similar oxidizable compounds in complex biosamples, the high resolution and selective detection of AA in a wide detection range remain a challenge^16^. Nanomaterials including ZnO nanowires on hierarchical graphene^17^, Fe_3_O_4_@gold (Au)-loaded graphenes^18^, multi-wall carbon nanotubes dispersed in polyhistidine^19^, and palladium (Pd) nanowire-modified graphene^20^ have been prepared for improving the selectivity of AA detection. While these nano-sensors showed a relatively wide detection range but performed with a restricted limit of detection. Other nanocomposites such as 3D graphene foam CuO nanoflowers^21^, over-oxidized polypyrrole (OPPy) and PdNPs/Au^22^, and graphene-supported platinum (Pt) nanoparticles^16^ have been used for ultrasensitive detection of AA but they performed with a restricted range of detection.

Nano-structuring of metal-grafted carbon nanostructures into conductive nanocomposites has provided high-caliber electrochemical sensors^23^. Graphene/polyaniline (PANI) nanocomposites with enhanced electrochemical properties and conductive characteristics have been developed for energy storage^24^, shielding of electromagnetic pollution^25^, electrocatalysis^26^ and particularly biosensing^27–30^. Incorporation of metal-NPs have also enhanced the electrical conductivity of the graphene/PANI composites^31^. However, majority of these metal-NPs/PANI structures are expensive and less available, with costly and time-consuming modification protocols, and have limited stability and reproducibility performance for reliable detection of small biomolecules in complex biological samples.

**Figure 1:**
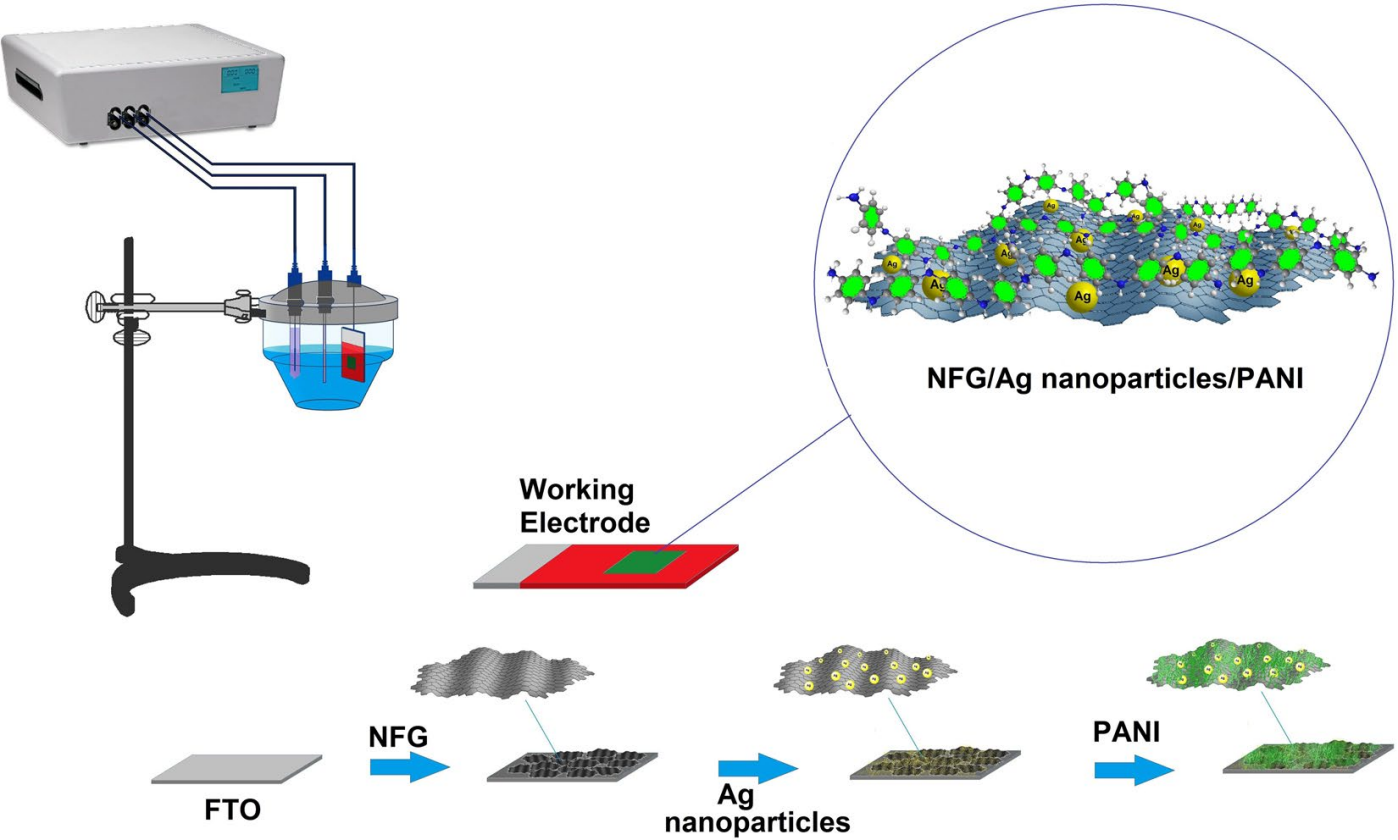
Schematic presentation of the synthesis procedure for metal nanoparticles (NPs)-grafted N-doped functionalized graphene (NFG)/polyaniline (PANI) nanocomposites on the fluorine doped tin oxide electrode (FTOE). The synthesis process of the nanocomposite consists of (1) coating of NFG on the FTOE substrate, (2) chronoamperometry of metal NPs on the NFG coated FTOE, and (3) cyclic voltammetric electropolymerization of PANI on AgNPs modified FTOE. The top right corner represents the final synthesized nanocomposite complex.

In this work, a new electrochemical sensor is developed for very low cost, highly sensitive and selective detection of AA in a wide detection range by an optimized sandwich arrangement of grafted silver nanoparticles (AgNPs) and nanostructured polyaniline (PANI) nanocomposite on nitrogen-doped functionalized graphene (NFG) electrode (Fig. 1). The biophysical properties and electrochemical activities of the NFG/AgNPs/PANI for AA oxidation were optimized to achieve a reproducible and stable sensing performance in biological samples. The results demonstrate that the presented nanocomposite exhibited highly conductive performance, suitable electrocatalytic activity and stable electron transfer kinetics towards the oxidation of AA. The selective detection of AA in the presence of Glu, DA, and UA is demonstrated with the sensitivity range of 28.9 to 280.5 mM.µA^−1^, detection limit of 8 µM, and a linear response range of 10–11,460 µM AA, applicable for both clinical healthcare and food safety applications.

## Results and Discussion

### Characterization of NFG/AgNPs/PANI nanocomposite

**Figure 2:**
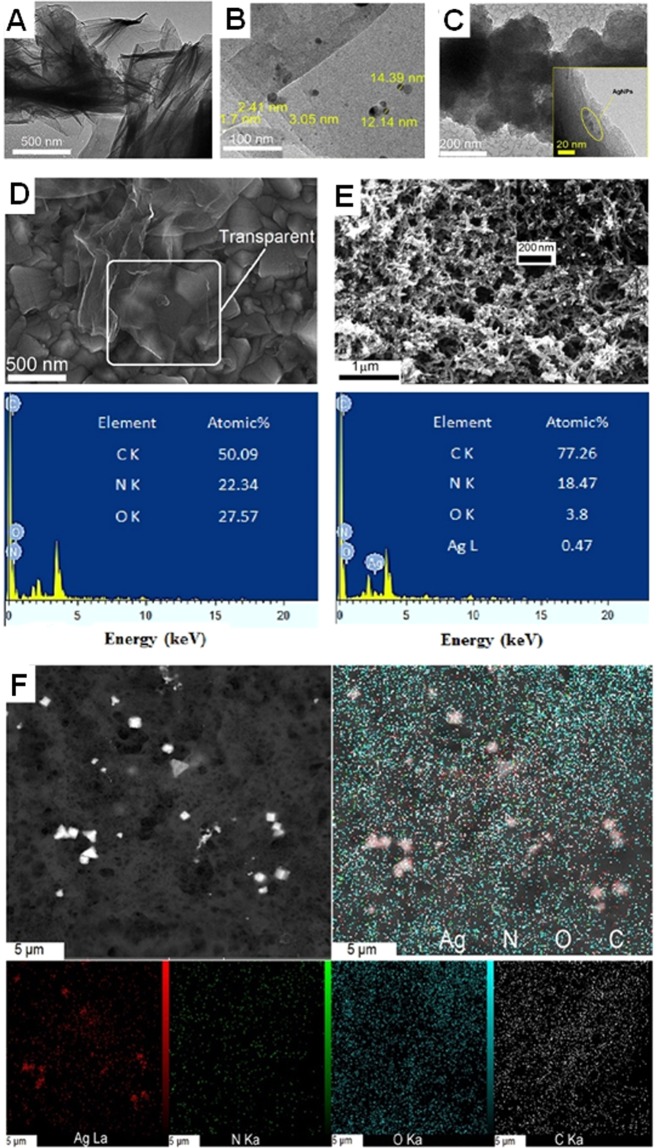
Characterization of the nanocomposite material coated on the FTOE. The ultrastructure of **(A)** the NFG sheets, **(B)** silver nanoparticles (AgNPs)-decorated graphene surface by applying the potential durations of 1 s and 90 s for the potentials of −0.40 V and 0.34 V, respectively, and **(C)** NFG/Ag/ PANI. The field emission scanning electron microscope (FESEM) micrographs and energy-dispersive X-ray spectroscopy (EDX) analysis of **(D)** NFG, and **(E)** NFG/Ag/PANI, respectively. **(F)** The mapping analysis of NFG/Ag/PANI surface which indicates the elemental distribution of the coated material on the substrate.

The morphologies and ultra-structures of NFG (A), NFG/Ag (B), and NFG/Ag/PANI (C) are shown in Fig. 2A–C. The transmission electron microscopy (TEM) images confirm the successful formation, morphology and structure of the nanocomposite material. Consistent with previous data^32–34^, the transparent graphene sheets with flake-like wrinkles remain stable under the electron beam exposure (Fig. 2A). The graphene sheets are sporadically decorated with AgNPs (size of ~2–15 nm) by applying optimized electrodeposition potential of −0.4 and 0.34 V and with the optimal potential durations of 1 s and 90 s, respectively (Fig. 2B). The surface polymerization accumulated clusters of the PANI on the graphene surface and gradually formed the thin PANI film covering the entire surface of the graphene.

The electropolymerization was completed and optimized successfully for AgNPs- modified graphene electrode (Fig. S1 and see SI.1 for details). Three redox peaks were detected based on the results of aniline electropolymerization (Fig. S2). The first peaks (a and a′) are related to the formation of cation-radicals; the second peaks (b and b′) result from production of the by-products and intermediates; and the last peaks (c and c′) are relevant to propagation of the polymer chains (Fig. S3)^35^. The formation of pellet/flake-like structure is an indication of the growth of PANI layer over the NFG (Fig. 2C). The transparent edges and black regions represent the graphene sheets and PANI in the NFG/Ag/PANI composite, respectively.

Energy-dispersive X-ray spectroscopy (EDX), field emission scanning electron microscope (FESEM), and mapping analysis were performed to determine surface chemical compositions of the NFG and NFG/Ag/PANI and provided topographical and elemental information of the surface (Fig. 2D–F). The FESEM data show the change of surface uniformity of the graphene nanosheets as a result of the AgNPs and PANI coating of the surfaces (Fig. 2E). The EDX analysis demonstrates the spectra that presents the peaks corresponding to the elemental composition of the coating on the surface. The EDX chemical characterization verifies the existence of carbon (C), oxygen (O), nitrogen (N), and Ag on the surface (Fig. 2D,E). Elemental mapping analysis shows the spatial distribution of elements in a sample and presents a complete 2D depiction of internal chemical zonation within a mineral. Fig. 2F is related to C, O, N, and Ag elements mapping and shows the uniform distribution of AgNPs on the electrode.

The results of Fourier-transform infrared spectroscopy (FTIR) spectra and Raman spectroscopy analysis were obtained at different steps of synthesizing AgNPs-grafted nitrogen-doped functionalized graphene nanostructured PANI nanocomposites. The results of FTIR data confirm the existence of various functional groups and chemical bonds in the specimen while the Raman analysis verifies that the composition of the coated layer relies on the content of carbon and conductive polymers. Both techniques in combination confirmed the successful modification of the electrodes, as demonstrated in our previous study^36^. The spectra of NFG/Ag/PANI was similar to NFG/PANI because the deposition of metal particles is not associated with new sets of binding among the particles or between the nanoparticles and two top and underlying layers. The spectrum bonds of PANI and NFG/PANI are noisy in surface-based diffuse reflectance spectroscopy (DRS)-based FTIR technique which inhibited the detection of potential effect of AgNPs on the peak intensity.

### Electrochemical characteristics of NFG/AgNPs/PANI nanocomposite

**Figure 3:**
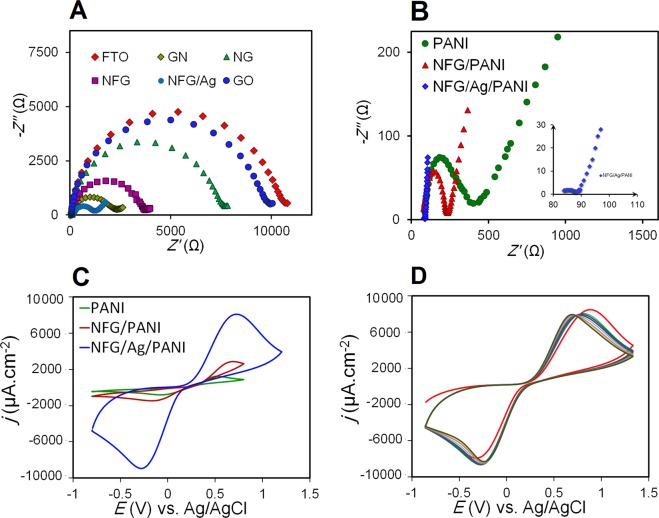
(**A**) Nyquist plots for the bare electrode of FTO and different stages of electrode functionalization such as graphene oxide (GO), functionalized GO with N-hydroxysuccinimide (NHS)/ 1-Ethyl-3-(3 dimethylaminopropyl) carbodiimide hydrochloride (EDC) (GN), N-doped graphene (NG), NFG, and NFG/Ag. **(B)** Nyquist plots and **(C)** cyclic voltammograms (CVs) for the PANI, NFG/PANI, NFG/Ag/PANI, respectively, on the FTOE surface and the inset in (B) is magnified Nyquist plot of NFG/Ag/PANI. All measurements were conducted in 0.01 M PBS (pH 7.4) containing 5 mM K_3_Fe(CN)_6_. **(D)** The CVs with seven cycles for the NFG/Ag/PANI on the FTOE surface with high stability performance.

The Nyquist plots and Cyclic voltammetry (CV) curves were obtained at different stages of electrode coating and functionalization, conducted in 0.01 M phosphate-buffered saline (PBS) (pH 7.4) containing 5 mM K_3_Fe(CN)_6_ (the electrochemical probe usually utilized to characterize the properties of biosensors). These two curves are used to investigate the electrochemical performance of NFG/AgNPs/PANI nanocomposite (Fig. 3A–C). The fluorine doped tin oxide electrode (FTOE) is known as a very low-cost substrate for sensing as it is at least 10 times cheaper than gold, however its intrinsic conductivity is very low. As shown in Fig. 3A, all components incorporated at different steps of our NFG substrate layer modification reduced the charge transfer resistance (*R*_*ct*_) respect to the bare FTOE and increased the electrode conductivity and surface area, providing a very low cost but highly conductive substrate for sensing applications. The presence of AgNPs on the graphene layer is clearly detectable (Fig. 3A). In the Nyquist plot, the semicircle portion at high frequencies corresponds the *R*_*ct*_. Due to the increase in the conductivity of the surface, the electron transfer conductivity with the Fe(CN)_6_^3−^ probe enhances and consequently forms the diffusion layer.

The PANI nanocomposites, due to the high porosity and surface area provided by its pellet/flake-like conductive construct, augmented the reaction kinetics and reduced the *R*_*ct*_ considerably (very smaller semicircle or kinetic control region). These nanocomposites do not have any redox peak in neutral media environment and act only as a surface conductive modifier (Fig. 3B). The critical modification of NFG/Ag/PANI-coated FTOE increased the conductivity of the electrode and reduced the *R*_*ct*_ dramatically. The primary parameters extracted from these curves, including the oxidation current density (*j*_ox_), solution resistance (*R*_*s*_), *R*_*ct*_, double-layer electrical capacitance (*C*_dl_), constant phase element (*CPE*), and Warburg diffusion element (*W*) are presented in Table S1. The *R*_*ct*_ was at its highest value of about 11,000 Ω for the very low cost FTOE, however the resistance dropped to about 6 Ω for the FTOE modified with the NFG/Ag/PANI composite, given the fact that the behaviour of the electrode is completely under control for mass transfer at low frequencies, surpassing all previous reports^37–43^. All these steps were fitted by [*R([RW]Q*)] equivalent circuit. *W* is related to mass-transfer controlled zone with a linear behaviour in low frequencies. *Q* is the *CPE* and observed when there is no ideal double-layer electrical capacitor behaviour. Applying the potential accumulates charges on the electrode surface and moves ions and polar molecules with the opposite charge towards the electrode to create the electrochemical capacitor. The charge accumulation and capacitance are elevated as a result of the coating of surface by conductive materials. According to Randles–Sevcik equation^44^, the active surface area obtained for the NFG/Ag/PANI modified FTOE is about 3.074 cm^2^ and is about 35 times higher than FTO bare electrode, indicating the higher electrochemical activity of the modified electrode.

There was a reasonable conformity between CV and Nyquist plot where the peak height in CV increased with a drop in the *R*_*ct*_ (Fig. 3B,C). The redox peaks at the NFG/Ag/PANI were significantly enhanced respect to other PANI and NFG/PANI coatings which may attribute to the excellent electron mediation of AgNPs/PANI and large surface area of NFG substrate; combined together they can accelerate the electron transfer with an enhanced current response.

The ratio of *R*_*ct*_ for the modified electrode (ME) to the *R*_*ct*_ for the bare electrode (BE) (*R*_*ct*_ ME/*R*_*ct*_ BE) in our NFG/Ag/PANI nanocomposite was much smaller than values reported in other studies (Table S2)^37–43^, clearly demonstrates that this nanocomposite can employ very low-cost substrates for making highly conductive electrodes. This remarkable conductivity opened avenues for the development of very low-cost and affordable electrodes but with excellent capability in nanocomposite coating and highly conductive performance.

Following the enhancement in conductivity performance of the NFG/Ag/PANI electrode, the stabilities of NFG, PANI, NFG/PANI, and NFG/Ag/PANI on FTOE surface were investigated. The successive CVs were applied in 0.01 M PBS containing 5 mM K_3_Fe(CN)_6_. The stability of the electrode was poor where the PANI layer was solely used for modification of the FTOE in the absence of NFG (Fig. S4B). The gradual dissolution of PANI in the aqueous medium (K_3_Fe(CN)_6_) may be the primary reason for instability of the electrode which resulted in a reduction in redox activity. However, the NFG deposited on the electrode was proven to be very stable (Fig. S4A). Also the electrode stability was improved significantly in the presence of both NFG and PANI, as demonstrated by the reasonable repeatability of seven CV cycles (Fig. S4C). The enhanced electrochemical stability could be related to the stronger interaction between the benzene ring and graphene sheets^45^. Ultimately, the stability of the coating increased dramatically in the presence of NFG/AgNPs/PANI due to the adhesive role of AgNPs between the upper PANI layer and lower graphene layer (Fig. 3D).

### Electrocatalytic reaction mechanism of AA with the NFG/Ag/PANI nanocomposite electrode

**Figure 4:**
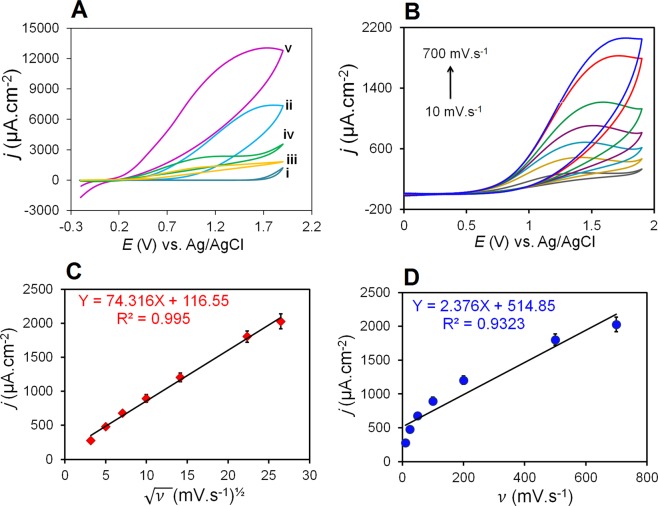
Electrocatalytic reaction mechanism of AA interaction with NFG/Ag/PANI nanocomposite. **(A)** CVs for different modification states: (i) NFG/Ag/PANI in 0.02 M PBS without ascorbic acid (AA), (ii) NFG, (iii) PANI, (iv) NFG/PANI, and (v) NFG/Ag/PANI in 0.02 M PBS with 25 mM AA at 100 mV.s^−1^. **(B)** CVs of NFG/Ag/PANI electrode at different scan rates of 10, 25, 50, 100, 200, 500, 700 mV.s^−1^ in 5 mM AA and 0.02 M PBS. The plot of peak current versus **(C)** the *ʋ*^½^ and **(D)** *ʋ*, respectively.

The sensing performance of the electrode coated with NFG/Ag/PANI nanocomposite was examined by detecting AA as an important vitamin for the synthesis and maintenance of human tissues^46^. CVs of the electrodes modified with NFG, PANI, NFG/PANI and NFG/Ag/PANI were measured in the absence and presence of AA (Fig. 4A). The results show no detectable peak in the PBS electrolyte in the absence of AA (i). However, the highest catalytic effect is detected in the presence of AA on the AgNPs-based nanocomposite (v) which is about two times, six times, and four times larger than the signal for NFG (ii), PANI (iii), and NFG/PANI (iv) modified electrodes, respectively.

The Randles–Sevcik equation was used to determine the diffusion or adsorption electrocatalytic mechanism of the AA with the NFG/Ag/PANI nanocomposite. The changes in the oxidation peak of AA were examined at different scan rates. The increase in scan rate from 10 to 700 mV.s^−1^ altered the oxidation peak potentials (Fig. 4B). A linear relationship was detected between the AA oxidation peaks current versus the square root of scan rate, indicating that the diffusion is the dominant reaction mechanism of AA on the electrode surface^47,48^ (Fig. 4C,D). Moreover, detecting the shift in the peak potential toward higher positive values for faster scan rate was another indication of the dominance of the AA diffusive mechanism on the electrode surface.

### Electrochemical oxidation of the AA with the NFG/Ag/PANI nanocomposite electrodes

All electrodes coated with NFG (i), PANI (ii), NFG/PANI (iii), and NFG/Ag/PANI (iv) showed amperometric responses proportional to the AA concentration (Fig. S5). To investigate the response of the electrodes to the concentration of the analyte and characterize the linearity performance of the nano-biosensor, the AA with 20 mM concentration was added to the electrodes stepwise in the volume of 12.5 µL every 200 s for the first 2,000 s of the experiment at the constant potential of 1.2 V (vs. Ag/AgCl) (Fig. S5A). The concentration of AA was then gradually increased to 200 mM for the next period of the experiment (2000–3000 s) where 12.5 µL of AA was added stepwise to the electrodes every 200 s. Finally, the experiment continued with adding the same concentration of 200 mM to the electrodes but this time with the 50 µL AA sample volume. The results show three different slopes for each of four NFG, PANI, NFG/PANI, and NFG/Ag/PANI electrodes which demonstrates the sensitivity of the sensor to both concentration and volume of AA samples. The results of peak current values against the AA concentrations tested in Fig. S5A are plotted in Fig. S5B. The results show that NFG electrode demonstrated a more intense variation of the electrical current against the spike of the AA but with a more weaving response respect to other PANI, NFG/PANI, and NFG/Ag/PANI electrodes tested under identical concentration of AA. This higher electrical current may attribute to active edges of the graphene and better interactions with AA. The changes in the electrical current for the PANI-coated or NFG/PANI modified electrode was not noticeable which may be due to the low stability of the PANI in aqueous environment. The NFG/Ag/PANI coating demonstrated a sustainable response compared to PANI and NFG/PANI modified electrodes, indicating that AgNPs promoted the charge transfer reactions of AA with the nanocomposite film, presented a significant role in the oxidation of AA, and enhanced the sensor stability during a long-time exposure of the electrode to aqueous environment under rigorous stirring conditions.

### Optimization of experimental parameters

**Figure 5:**
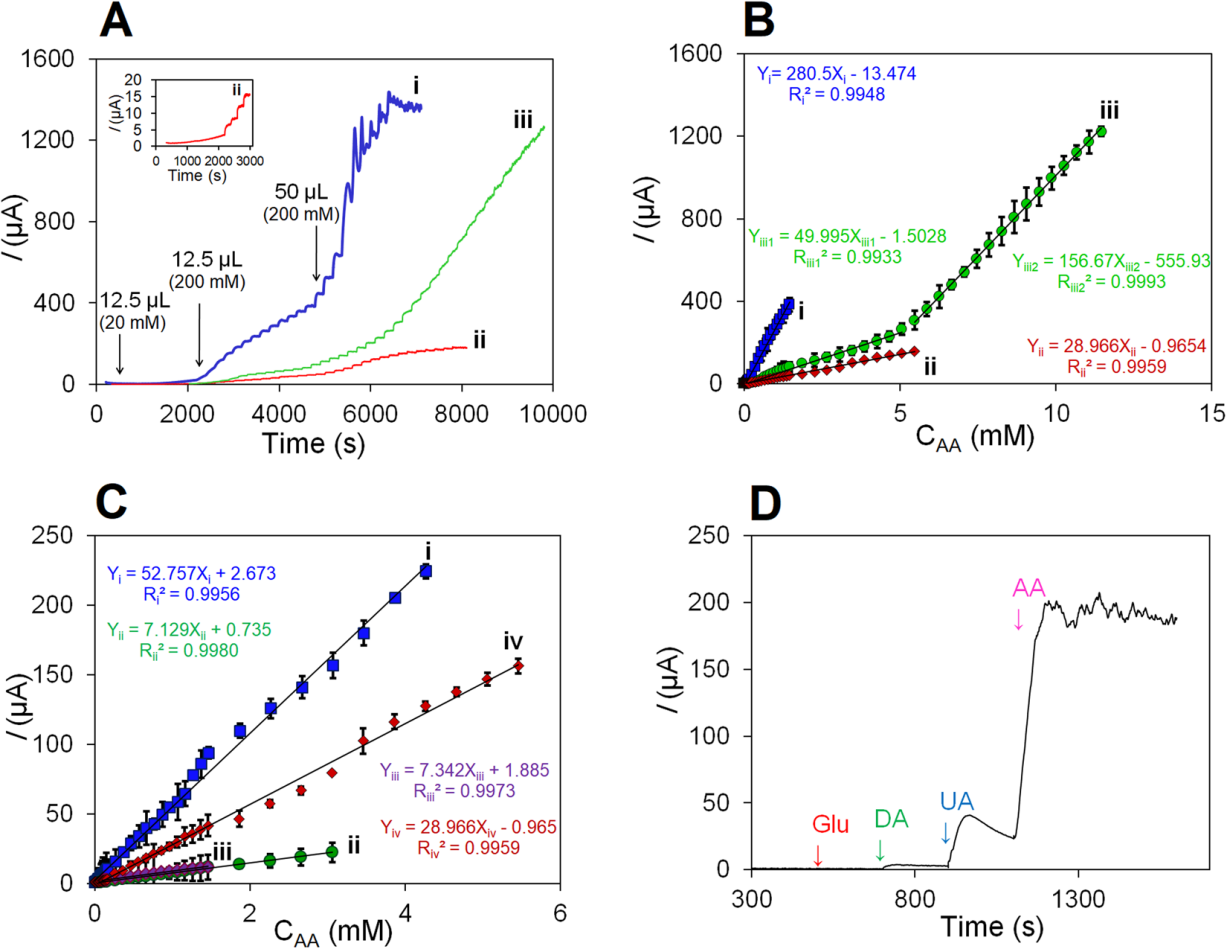
The amperometric response **(A)** and linear detection range **(B)** of the proposed nano-biosensor for detecting AA oxidation on NFG/Ag/PANI with different deposition duration of (i) 1 s and 30 s, (ii) 1 s and 90 s and (iii) 10 s and 90 s for *E*_1_ and *E*_2_, respectively. **(C)** The linear detection response of the nano-biosensor for detecting the AA oxidation for the (i) NFG, (ii) PANI, (iii) NFG/PANI, and (iv) NFG/Ag (with optimal deposition duration of 1 s and 90 s for *E*_1_ and *E*_2_)/PANI with n = 3 and RSD < 5% in 25 mL 0.01 M PBS (pH 7.4). **(D)** The selectivity of AA nanocomposite biosensor in the presence of three interferences of glucose (Glu), dopamine (DA), and uric acid (UA) in 0.02 M PBS at the applied potential of 1.2 V (vs. Ag/AgCl), in 0.01 M PBS (pH 7.4).

The oxidation peak potential of the AA catalysis was obtained by CA technique and used to determine the detection range of the NFG/Ag/PANI nano-biosensor (Fig. 5A). The size of AgNPs was shown to be very effective on sensitivity and linear response of the sensor to AA concentration. The amperometric response of the NFG/Ag/PANI electrode for detecting AA oxidation was examined for different AgNPs electrodeposition duration of 1 s and 30 s (i), 1 s and 90 s (ii) and 10 s and 90 s (iii) for *E*_1_ and *E*_2_, respectively (Fig. 5B). A higher electrical current was detected for the AgNPs electrodeposition duration of 1 s and 30 s due to higher surface area and smaller size of the NPs which caused a faster response to AA. However, the response for this electrodeposition duration was less linear because of lower quantity of AgNPs. These concentration curves revealed a reasonable linear response with a very wide detection range for the optimal electrodeposition duration of 1 s and 90 s for *E*_1_ and *E*_2_, respectively, on NFG/Ag/PANI coated electrode (Fig. 5B). Similar optimal electrodeposition duration of 1 s and 90 s for *E*_1_ and *E*_2_, respectively, was determined for NFG/Ag electrodes characterized by Nyquist plots (Fig. S6). The optimal size of AgNPs was further used to examine the sensitivity and detection range performances of the NFG/Ag/PANI and compare it with NFG (i), PANI (ii), NFG/PANI (iii) coated electrodes in their response to successive aliquots of increasing concentrations of AA (Fig. 5C). The results show that the peak currents of the AA detected on NFG/Ag/PANI with optimal AgNP size increased linearly with a higher sensitivity and wider detection range respect to the linear response of PANI and NFG/PANI electrodes. The AA detection limit of 8 µM (S/N = 3) and the linear detection range of 10–5,460 µM was obtained for the NFG/Ag/PANI sensor tested in the optimal coating and functionalization conditions (Table 1). The detection limit of the sensor (S/N = 3) and its linear range were compared to AA sensors comprising similar materials (Table 2). To the best of our knowledge, the linear range of this nanocomposite was wider than any other electrochemical sensor based on nanostructured materials reported in the literature.

**Table 1:** The detection limit and linear detection range of different modified electrodes used for AA detection.

Electrode materials	Detection limit (µM)	Linear range (µM)
NFG	10	10–4, 270
PANI	60	60–3, 060
NFG/PANI	100	100–1, 460
NFG/Ag (1, 30 s)/PANI	60	60–1,460
NFG/Ag (1, 90 s)/PANI	8	10–5, 460
NFG/Ag (10, 90 s)/PANI	50	50–11, 460

**Table 2:** The comparison of the proposed nanocomposite-based biosensor with other efforts reported in the literature.

Electrode materials	Detection limit (µM)	Linear range (µM)	Reference
PANI/PSS/Gr	5	100–1000	^45^
NG	2.2	5–1300	^51^
CoPc–MWCNTs	1	10–2600	^52^
AGCE/ASOD	2	5–400	^53^
PdNi/C nanomaterials	0.5	10–1800	^54^
MWCNT/CCE	7.71	15–800	^55^
Pt-Au hybrid	103	103–165	^56^
Chitosan-graphene	50	50–1200	^57^
OMC/Nafion	20	40–800	^58^
ZnO/RM	1.4	15–240	^59^
MBMOR/P	12.1	20–800	^60^
Pd/CNFs	15	50–4000	^49^
PMPy/Pd-nanoclusters	1000	50–1000	^61^
DB71	1	1–2000	^62^
BPPF_6_/CPE	8	10–3000	^63^
PPF/GNS	120	400–6000	^64^
PdNPs-GO	—	20–2280	^1^
G-BSA	25	50–3000	^65^
NG/Au/PANI	640	960–4660 and 5060–9860	^66^
NFG/Ag/PANI	8	10–11460	This work

### Selectivity investigation and AA assay in real sample

The AA selectivity of the NFG/Ag/PANI non-enzymatic sensor was examined by detecting the AA within known electroactive species of Glu, DA, and UA (Fig. 5D). These species are often electroactive in the positive potential region^16,49^. The AA concentration of 5 mM was detectable electrochemically in the presence of higher values of about 1.5 times (7 mM) more than AA for Glu, UA, and DA. Since the analyte AA exhibited a minimal interference from endogenous electroactive species, the sensor has commercialization capability for AA detection. The performance of the sensor was assessed in real sample of vitamin C tablet containing 500 mg of AA. The certain concentrations were prepared and tested by the sensor. The results were compared to the data from iodometry standard method (iodometric titration; the AA obtained from tablets was titrated with 0.01 M I^3−^ in the presence of starch glue as an indicator) (Table S3). The comparison of theoretical and experimental F-test confirmed a negligible difference between the measurement with our nano-biosensor and the iodometry method with the recovery of 98%. Ultimately, the stability of the NFG/Ag/PANI nanocomposite was investigated after 14 days storage in room temperature, where after this period, the electrode modified with the nanocomposite was used to detect AA at 7 mM concentration. The results show the detection efficiency of about 98.7% even after the 14 days storage of the nanocomposite-coated electrode in room temperature (Fig. S7).

In summary, the AgNPs-grafted NFG-nanostructured PANI nanocomposite were prepared and optimized on low-cost FTOE substrates. NFG was placed on the FTOE surface as the substrate, meanwhile AgNPs were sandwiched between the NFG and PANI. The fabricated NFG/Ag/PANI electrodes produced a very large surface area, excellent conductivity and electrocatalytic activity, and high stability for the detection of AA in a linear and wide detection range performance. The approach proposed for making this nano-biosensor proved that the linear detection range and the detection limit of the non-enzymatic sensors could be improved considerably by adjusting the size, density and morphology of nanoparticles. This nano-biosensor was further used for the detection of AA in the presence of interfering DA, Glu, and UA signals as well as in vitamin C tablets, demonstrating its promising performance for determining the AA concentration in biological samples.

## Experimental

### Reagents and Materials

The graphite powder (spectroscopic grade, particle size ≤ 40 µm), potassium nitrate (KNO_3_), silver nitrate (AgNO_3_), sodium nitrate (NaNO_3_), potassium permanganate (KMnO_4_), sulfuric acid (H_2_SO_4_, purity 98%), and potassium ferricyanide (K_3_Fe(CN)_6_) were purchased from Merck Inc. Other chemicals of 1-Ethyl-3-(3 dimethylaminopropyl) carbodiimide hydrochloride (EDC, purity 98%), N-hydroxysuccinimide (NHS, purity 98%), PBS, dimethylformamide (DMF), and aniline (purity 99%) were purchased from Sigma-Aldrich. All other reagents and chemicals used for electrochemical experiments were of analytical grade and utilized without any further purification. The deionized (DI) water for solution preparation was provided from a Millipore Sigma (20 MΩ, Millipore, USA). FTOE as a very low cost substrate was purchased from Solaronix. All glassware was autoclaved and kept in an appropriate place to eliminate any cross-contamination.

### Instrument and measurement

Morphology of AgNPs was determined by the FESEM images. The EDX data and mapping analysis were obtained by Carl Zeiss FESEM instrument. The TEM micrographs were obtained using the FEI Tecnai TF20 at 200 kV. All electrochemical studies were performed using a Potentiostat/Galvanostat model of Autolab PGSTAT 30 (Echo chemie, B. V., Netherlands) by Nova 1.11 software. CV and chronoamperometry (CA) techniques were used for electrochemical measurements. Electrochemical impedance spectroscopic (EIS) measurements were accomplished within the frequency range of 10^5^–10^−1^ Hz, with the potential amplitude of 14 mV around the open circuit potential (*E*_ocp_). The conventional three-electrode system consisting of a modified FTO as the working electrode, a platinum rod as the auxiliary electrode and an Ag|AgCl|3 M KCl as the reference electrode was used throughout the experiments. Error bars were established by 3 independent electrodes.

### Preparation of AgNPs/PANI nanocomposite over functionalized graphene electrode

Briefly, a few layers of NFG as a conductive substrate was prepared by the modified Hummer method and functionalized with −COOH functional group by EDC/NHS^36^. The functionalized graphene oxide (GO) was reduced by incorporating the DMF and nitrogen doped in DI water. The resulted NFG electrode was decorated with Ag nanoparticles by dual potential CA technique to enhance the surface area and improve adhesion of the layers. PANI was then electropolymerized on nanoparticle-decorated electrodes by CV technique to increase electron transfer conductivity^50^ and coat the AgNPs to inhibit electrode oxidation (See Supporting Information SI.1 for details).

### Detection of real samples

The performance of the nano-biosensor was assessed in real samples, where vitamin C tablets containing 500 mg of AA were analysed. Three vitamin C tablets were weighted and then milled. The certain concentrations of vitamin C were prepared and spiked into the 0.01 PBS (pH 7.4) at specified intervals, and efficacy of the nano-biosensor for AA detection was evaluated.

## Supplementary information

Sandwich-structured nanoparticles-grafted functionalized graphene based 3D nanocomposites for high-performance biosensors to detect ascorbic acid biomolecule
