# Supplementary material for: Sandwich-structured nanoparticles-grafted functionalized graphene based 3D nanocomposites for high-performance biosensors to detect ascorbic acid biomolecule
Source: Sci Rep. 2019 Feb 4;9:1226. doi: 10.1038/s41598-018-37573-9 (PMC6362002; doi:10.1038/s41598-018-37573-9)
Supplement: Supplementary file 1 — Sandwich-structured nanoparticles-grafted functionalized graphene based 3D nanocomposites for high-performance biosensors to detect ascorbic acid biomolecule [file 41598_2018_37573_MOESM1_ESM.docx]

**Sandwich-structured nanoparticles-grafted functionalized graphene based 3D nanocomposites for high-performance biosensors to detect ascorbic acid biomolecule**

Razieh Salahandish, Ali Ghaffarinejad, Seyed Morteza Naghib, Asghar Niyazi, Keivan Majidzadeh-A, Mohsen Janmaleki and Amir Sanati-Nezhad

**SI**: Supporting Information

## **SI.1 Synthesis and characterization of the nanocomposite**

## **Synthesis of functionalized graphene**

Graphite oxide was synthesized by modified Hummer method^1^. Briefly, graphite powder (2 g) was sequentially mixed with the concentrated sulfuric acid (H_2_SO_4_) (50 mL) and sodium nitrate (NaNO_3_) (2 g). After stirring in an ice bath, [potassium permanganate](https://en.wikipedia.org/wiki/Potassium_permanganate) (KMnO_4_) (6 g) was gradually added to the mixture exothermically up to 35 °C and stirred for 2 hrs. Deionized (DI) water (100 mL) was then added to the reaction vessel at 90 °C and stirred for 30 min. The reaction was stopped by adding DI water (200 mL) and hydrogen peroxide (30%, 10 mL).

In the first stage of graphene oxide functionalization, the synthesized graphene oxide (0.1 g) was suspended in 10 mL 1-Ethyl-3-(3 dimethylaminopropyl) carbodiimide hydrochloride (EDC) (400 μM) and N-hydroxysuccinimide (NHS) (100 μM) (prepared with DI water)^2^. The resulting solution was mixed for 4 hrs at room temperature, separated by centrifugation, and washed several times to remove residual EDC and NHS. The acquired solid material was dried at 45 °C. In the second stage of graphene functionalization, the resulting material in the first stage (5 mg) was added to dimethylformamide (DMF) (10 mL) and heated to the boiling point for 1 hr. The extracted product was washed with DI water to produce nitrogen-doped functionalized graphene (NFG). All products obtained in each stage were dispersed in DI water (2.5 mg mL^-1^) ^1^.

## **Graphene-based modification of FTOE electrode**

To modify the electrode surface, fluorine doped tin oxide (FTO) glass plates (8 Ω resistance) with the surface area of 0.25 cm^2^ were exposed to sequential ultrasonic cleaning in acetone, ethanol, isopropanol and DI water for 10 min. FTO sheets were dried under the flow of argon gas. The NFG suspended in DI water was then deposited (5, 10, 20, 40, and 50 µL) on the FTO surface by cast coating (the optimal volume of NFG was determined to be 40 µL) and allowed to dry at 45 °C.

## **Electrodeposition of silver nanoparticles and electropolymerization of aniline**

Silver nanoparticles (AgNPs) were electrodeposited on the electrode after the deposition of graphene on the FTOE. To investigate the redox potential of AgNPs on the FTOE, two cyclic voltammograms were applied on the electrode surface in 1 mM silver nitrate (AgNO_3_) and 0.1 M potassium nitrate (KNO_3_) with the similar scan rate of 50 mV.s^-1^. AgNPs were electrodeposited by dual potential pulse chronoamperometry (*E*_1_ and *t*_1_; -0.4 V, 1, 2, 3, 5, 10 s; *E*_2_ and *t*_2_; 0.34 V, 90 s, and *E*_1_ and *t*_1_; -0.4 V, 1 s (optimum duration); *E*_2_ and *t*_2_; 0.34 V, 30, 60, 90, 120, 150 s). The electrochemical impedance spectroscopy (EIS) testing was performed to determine electrodeposition duration^3,4^. The electrochemical cell consisted of conventional three-electrode system. The Ag|AgCl|3M KNO_3_ and Pt rod were used as the reference, and counter electrodes of the modified FTOE (working electrode), respectively. For electrodeposition of the PANI on graphene/NPs-treated FTOE, the optimal number of 20 (between of cycles 5, 10, 20, 30, and 40) successive cyclic voltammograms were applied on the electrode surface in a solution of 0.03 M aniline monomer and 0.5 M H_2_SO_4_. This process was performed at the potential range of -0.4 to 1.2 V and with the scan rate of 30 mV.s^-1^ ^5^.

**SI.2 Results and discussion**

The cyclic voltammetry analysis of the electrodeposited silver (1 mM) on the FTOE showed that the potential of the reduction peak for silver shifts toward positive values in the second cycle (250 mV), opposed to negative shifts detected in the first cycle (20 mV) (**Fig. S1**). The peak shift indicated that AgNPs were deposited on the FTOE surface at the end of the first cycle while the silver deposition occurred with a lesser energy in the second cycle. The dual potential pulse technique of the chronoamperometry was used to adjust potentials for two pulses. The electrical potential was selected more negative for the first pulse opposed to the electrical potential of the reduction peak. However, the electrical potential was chosen more positive for the second pulse comparing to the potential of the reduction peak. Based on CV curves, the potentials for the first and second pulses were selected to be -0.40 and 0.34 V, respectively. However, the selection of different potential duration resulted in different sizes of AgNPs.

**Figure S1.** The cyclic voltammetry (CVs) of the electrodeposited silver (Ag) in the presence of 1 mM silver nitrate (AgNO_3_) and 0.1 M potassium nitrate (KNO_3_) on the fluorine doped tin oxide electrode (FTOE) at 50 mV.s^-1^ sweep rate.

**Figure S2.** The electropolymerization of the aniline in the presence of 0.5 M sulfuric acid (H_2_SO_4_) containing 0.03 M aniline monomer (scan rate: 30 mVs^-1^). Note that a-aʹ, b-bʹ and c-cʹ represent cation-radicals, by-products or intermediates, and polymer chain propagation, respectively.

1) Formation of cation-radicals

2) Production of by-products intermediates

3) Formation of polymer chain propagation

**Figure S3**. The polymerization stages of PANI.

**Table S1.** Important electrochemical parameters during the systematic optimization of FTOE.

| Working electrode | *j_ox_*  (mA cm^-2^) | *R_s_*  (Ω) | *R_ct_*  (Ω) | *CPE*.Y0  (µMho) | *C*_dl_  (µF) | *W*.Y0  (µMho) | equivalent circuit | *R_ct_* ME/ *R_ct_* BE (%) |
| --- | --- | --- | --- | --- | --- | --- | --- | --- |
| FTO | 0.38 | 95 | 11,000 | 2.80 | 6 | 2,390 | [R([RW]Q)] | - |
| NFG | 0.63 | 102 | 3,650 | 2.74 | 4 | 3,690 | [R([RW]Q)] | 33.18 |
| NFG/Ag | 0.85 | 120 | 1050 | 5.27 | 17 | 2,550 | [R([RW]Q)] | 9.54 |
| PANI | 9.22 | 102 | 300 | 10.3 | 64 | 4,550 | [R([RW]Q)] | 2.72 |
| NFG/PANI | 10.36 | 84 | 150 | 35.2 | 2,591 | 4,660 | [R([RW]Q)] | 1.36 |
| NFG/Ag/PANI | 13.33 | 83 | 6 | 21,500 | 24,233 | 109,000 | [R([RW]Q)] | 0.05 |

*j_ox_*; oxidation current density, ME; modified electrode, BE; bare electrode

**Table S2**. Comparison of the electrochemical performance of metal nanoparticles-grafted functionalized graphene (MFG)/PANI nanocomposites developed in this work respect to the recently reported counterparts developed based upon graphene/PANI and graphene/carbon nanotubes/PANI.

| BE | ME | *R_ct_* of BE (Ω) | *R_ct_* of ME (Ω) | *R_ct_* of PANI (Ω) | *R_ct_* ME /  *R_ct_* BE (%) | *R_ct_* ME /  *R_ct_* PANI (%) | Reference |
| --- | --- | --- | --- | --- | --- | --- | --- |
| GCE | GR/PANI | 4,000 | 400 | - | 10.00 | - | ^6^ |
| Pt | GNS/MWCNT/PANI | 1.48 | 0.38 | 5.4 | 25.67 | 7.04 | ^7^ |
| Pt | PANI/8 wt% graphene | - | 11.49 | 64.5 | - | 17.81 | ^8^ |
| GCE | GR/PANI | 4,000 | 60 | - | 1.50 | - | ^9^ |
| GCE | ERGNO/PAN | 647 | 275 | 346 | 42.50 | 53.48 | ^10^ |
| ITO | GrO/PANI | - | 5,432 | 38,380 | - | 14.15 | ^11^ |
| GCE | PANIw/graphene | 800 | 20 | - | 2.5 | - | ^12^ |
| FTO | **MFG/PANI** | **11,000** | **6** | **290** | **0.05** | **2.07** | **Present work** |

BE; bare electrode, ME; modified electrode

**Figure S4.** The CVs with seven cycles for the (A) NFG and (B) PANI and (C) NFG/PANI on the FTOE surface. All measurements were conducted in 0.01 M PBS (pH 7.4) containing 5 mM K_3_Fe(CN)_6_. The CV trend for PANI declined but remained relatively unchanged for NFG.

**
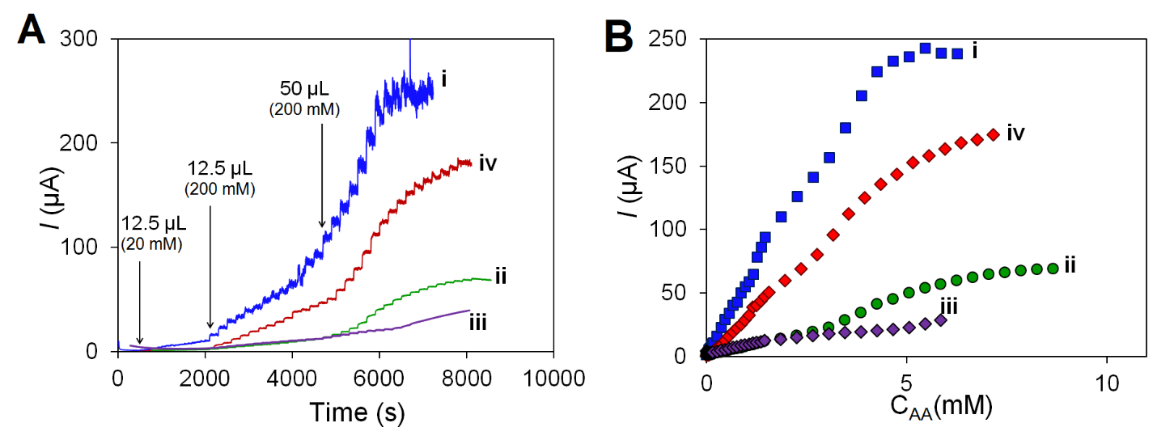
**

**Figure S5.** The amperometric response (A) and total detection range (B) of the sensor for detecting the AA oxidation for i) NFG, ii) PANI, iii) NFG/PANI, iv) NFG/Ag (with deposition duration of 1 s and 90 s for *E*_1_ and *E*_2_)/PANI with n = 3 and RSD < 5 in 25 mL 0.01 M PBS (pH 7.4).

The effect of NPs size on the electrochemical performance is shown in **Fig. S6**. The results showed that the silver concentration increased on the electrode by increasing the potential duration. The higher electrodeposition duration, however, led to an increase in the size of NPs and a reduction in the effective area for the exchange of electrons with Fe(CN)_6_^3-^ probe. The increase in the semicircle diameter of the Nyquist plot also confirmed that the size of NPs deteriorated the charge transfer.


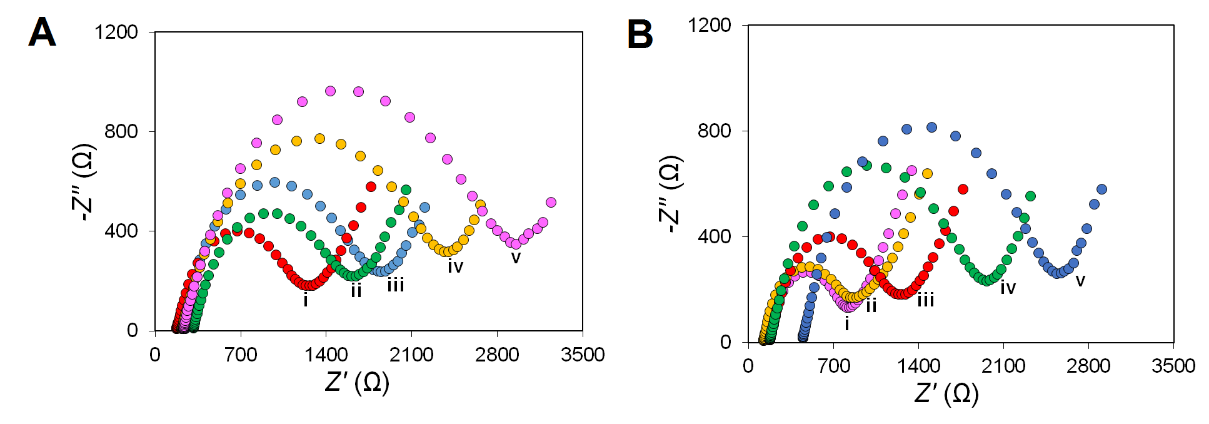


**Figure S6.** Nyquist plots for NFG/Ag at different electrodeposition durations of AgNPs; (A) a) 1 s and 90 s, b) 2 s and 90 s, c) 3 s and 90 s, d) 5 s and 90 s and e) 10 s and 90 s; (B) a) 1 s and 30 s, b) 1 s and 60 s, c) 1 s and 90 s, d) 1 s and 120 s and, e) 1 s and 150 s for *E*_1_ = -0.40 and *E*_2_ = 0.34, respectively, in 0.01 M PBS (pH 7.4) containing 5 mM K_3_Fe(CN)_6_.

**Table S3.** Results of detecting the real sample (vitamin C tablet containing 500 mg of AA) by NFG/AgNPs/PANI sensor (tested with potential durations of 1 s and 90 s for potentials of -0.4 and 0.34 V, respectively). The results is compared to the data obtained from the iodometry method.

|  | Calculated amount of AA  (mg) | RSD  (%) | Recovery  (%) | S^2^ | F-test (S^2^_1_/S^2^_2_) |
| --- | --- | --- | --- | --- | --- |
| Iodometry method | 490 ± 10 | 2 | 98 | 100 |  |
| NFG/AgNPs/PANI | 479 ± 4 | 0.8 | 96 | 16 |  |
| Experimental F (EF) |  |  |  |  | 6.25 |
| F_2, 2_ |  |  |  |  | 19 |
| EF< F_2, 2_, No significant difference | | | | | |

**Figure. S7.** Stability of the NFG/Ag/PANI nanocomposite, stored for seven and 14 days in room temperature, were tasted for the detection of 7 mM AA. The results show that the nao-biosensor preserved its detection efficiency for about 98.7% and remained very stable even after 14 days storage.


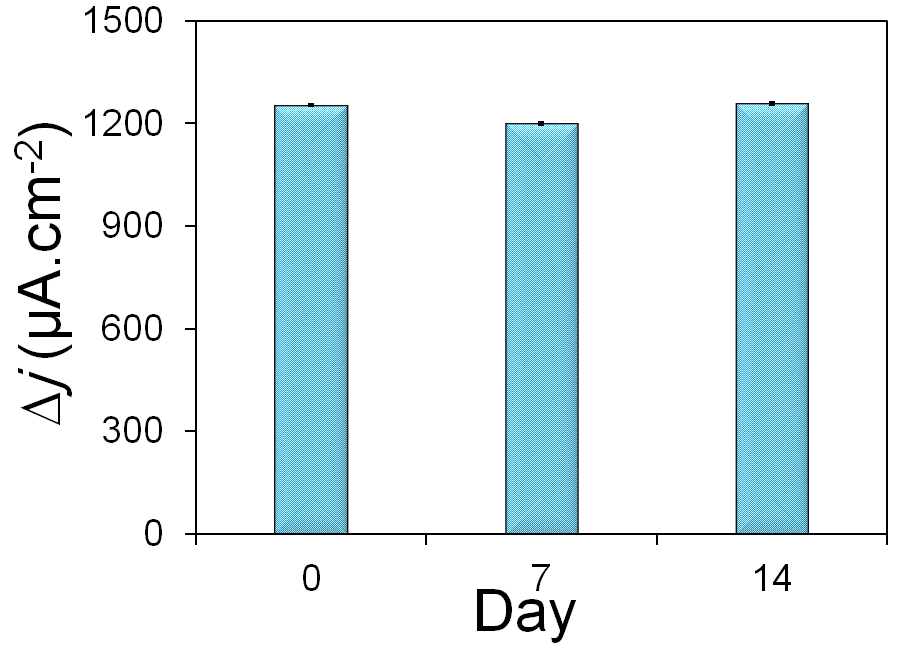


## **References**

1 Li, H., He, J., Li, S. & Turner, A. P. Electrochemical immunosensor with N-doped graphene-modified electrode for label-free detection of the breast cancer biomarker CA 15-3. *Biosensors and Bioelectronics* **43**, 25-29 (2013).

2 Cernat, A., Tertiș, M., Păpară, C. N., Bodoki, E. & Săndulescu, R. Nanostructured platform based on graphene-polypyrrole composite for immunosensor fabrication. *Int. J. Electrochem. Sci* **10**, 4718-4731 (2015).

3 Ustarroz, J., Gupta, U., Hubin, A., Bals, S. & Terryn, H. Electrodeposition of ag nanoparticles onto carbon coated tem grids: A direct approach to study early stages of nucleation. *Electrochemistry Communications* **12**, 1706-1709 (2010).

4 Geboesa, B. *et al.* Influence of the Morphology of Electrodeposited Nanoparticles on the Activity of Organic Halide Reduction. *Chemical Engineering* **41** (2014).

5 Darowicki, K. & Kawula, J. Impedance characterization of the process of polyaniline first redox transformation after aniline electropolymerization. *Electrochimica Acta* **49**, 4829-4839 (2004).

6 Liu, S. *et al.* A novel label-free electrochemical aptasensor based on graphene–polyaniline composite film for dopamine determination. *Biosensors and Bioelectronics* **36**, 186-191 (2012).

7 Al-Bahrani, M. R. *et al.* Highly efficient dye-sensitized solar cell with GNS/MWCNT/PANI as a counter electrode. *Materials Research Bulletin* **59**, 272-277 (2014).

8 He, B., Tang, Q., Wang, M., Ma, C. & Yuan, S. Complexation of polyaniline and graphene for efficient counter electrodes in dye-sensitized solar cells: Enhanced charge transfer ability. *Journal of Power Sources* **256**, 8-13 (2014).

9 Fan, Y., Liu, J.-H., Yang, C.-P., Yu, M. & Liu, P. Graphene–polyaniline composite film modified electrode for voltammetric determination of 4-aminophenol. *Sensors and Actuators B: Chemical* **157**, 669-674 (2011).

10 Du, M., Yang, T., Li, X. & Jiao, K. Fabrication of DNA/graphene/polyaniline nanocomplex for label-free voltammetric detection of DNA hybridization. *Talanta* **88**, 439-444 (2012).

11 Radhapyari, K., Kotoky, P., Das, M. R. & Khan, R. Graphene–polyaniline nanocomposite based biosensor for detection of antimalarial drug artesunate in pharmaceutical formulation and biological fluids. *Talanta* **111**, 47-53 (2013).

12 Bo, Y., Yang, H., Hu, Y., Yao, T. & Huang, S. A novel electrochemical DNA biosensor based on graphene and polyaniline nanowires. *Electrochimica Acta* **56**, 2676-2681 (2011).
